# Clinical and Epidemiological Profile, Mortality, and Associated Factors Among Paediatric Burn Patients in Western Rajasthan, India

**DOI:** 10.7759/cureus.111793

**Published:** 2026-06-30

**Authors:** Shaheed Khan, Avdhesh K Sharma, Shaitan Singh Rathore, Mahendra Choudhary, Sweta Singh

**Affiliations:** 1 General Surgery, Dr. Sampurnanand Medical College, Jodhpur, IND

**Keywords:** factors associated with mortality, haemoglobin, hypokalaemia, low- and middle-income countries, paediatric burns, pseudomonas aeruginosa, total body surface area

## Abstract

Background

Paediatric burn injuries continue to be a significant source of morbidity and mortality in low- and middle-income countries (LMICs), with outcomes influenced by the severity of the injury, systemic healthcare constraints, and socio-environmental risk factors. This study aimed to assess the clinico-epidemiological characteristics, mortality burden, and factors associated with mortality among paediatric burn patients treated in a tertiary care facility in Western Rajasthan, India.

Materials and methods

A prospective, longitudinal, hospital-based observational study was performed in the burn unit and the surgical wards of Mahatma Gandhi Hospital, Jodhpur, from April 2025 to December 2025. A total of 100 paediatric burn patients aged 18 years or younger with acute burn injuries were included, excluding individuals with healed burns or pre-existing systemic comorbidities. Demographic, clinical, laboratory, and microbiological data were documented utilising a structured case report form. Statistical analysis was conducted using MedCalc® Statistical Software version 22.0 (MedCalc Software Ltd., Ostend, Belgium); a p-value of less than 0.05 was deemed significant.

Results

Among 100 enrolled patients, 64% (64/100) were men, and 70% (70/100) were aged one to five years. Scald burns were predominant (64/100, 64%), with the head and neck being the most frequently affected areas (63/100, 63%). The mortality rate was 12% (12/100). Factors associated with mortality were total body surface area (TBSA) of >40% (odds ratio {OR}=9.60; p=0.001), haemoglobin of <9.5 g/dL at 48 hours (p<0.001), serum potassium of <3.4 mEq/L (p=0.044), *Pseudomonas aeruginosa* colonisation by day 6 (p=0.023), and hospital stay of less than five days (p=0.004). Receiver operating characteristic (ROC) analysis identified a TBSA threshold of 30% as the factor with the highest discriminatory performance for mortality (area under the curve {AUC}=0.973).

Conclusion

Greater burn surface area, early anaemia, hypokalaemia, and *Pseudomonas aeruginosa* colonisation were associated with mortality. Prioritising immediate fluid resuscitation, infection prophylaxis, electrolyte normalisation, and caregiver training is essential for enhancing outcomes in resource-constrained environments.

## Introduction

Burn injuries are among the most severe forms of trauma and trigger a systemic inflammatory response, characterised by immunological dysregulation, fluid and electrolyte imbalances, neuroendocrine disturbances, and hypermetabolism, which affects many organ systems and may continue long after the original trauma [1].

Paediatric burn injuries produce more severe physiological effects compared to adults, due to children's thinner dermal layers, higher body surface area-to-mass ratios, immature thermoregulatory mechanisms, and limited physiological reserves [2]. Thus, these injuries may demand customised paediatric therapies including structured feeding programmes, physiotherapy, infection management, and psychological support that prevent more harm and facilitate recovery [3,4].

More than 95% of burn-related deaths occur in low- and middle-income countries (LMICs), where children are disproportionately affected by burn injuries [4]. Most scald injuries among children under five years of age in India are caused by hot liquids and open flames and are frequently associated with unsafe household environments and inadequate caregiver supervision [5]. Inadequate access to specialised child burn facilities, delayed referrals, dependence on conventional healing methods, and a lack of first-aid expertise all contribute to the problem [6,7]. Common clinical consequences that have a major impact on outcomes include anaemia, hypokalaemia, oliguria, sepsis, and malnutrition [8-10]. These difficulties have been reported in studies conducted in Ethiopia, Ghana, and other LMICs, which highlight the importance of prompt intervention, early fluid resuscitation, and infection control as critical factors influencing survival [11-13]. Furthermore, the psychological effects, the possibility of hypertrophic scarring, and the difficulties with social reintegration continue to be significant post-recovery issues, particularly for children [14].

The primary objective of this study was to assess mortality and factors associated with mortality among paediatric burn patients treated at a tertiary care institution in Western Rajasthan. Secondary objectives were to describe the clinico-epidemiological profile, burn characteristics, microbiological findings, and treatment-related outcomes in the study population. This prospective study, in contrast to retrospective or registry-based analyses, collects real-time clinical and laboratory data on important mortality factors, including total body surface area (TBSA), haemoglobin levels, electrolyte imbalances, microbial colonisation, and early renal failure, that facilitate more precise and rapid identification of high-risk patients [15]. The project seeks to optimise the utilisation of scarce healthcare resources by identifying high-risk indicators and outcome modifiers, enabling the development of personalised treatment algorithms and informing public health policies, including targeted burn prevention initiatives, community first-aid training programmes, and referral guidelines for specialised burn units, tailored to local population needs [16].

Although several studies have evaluated paediatric burn injuries in India, most have primarily described epidemiological patterns or general risk factors, with limited prospective evidence integrating clinical, laboratory, microbiological, and outcome-related variables from Western Rajasthan. Regional differences in burn mechanisms, healthcare access, referral practices, and microbial profiles may influence patient outcomes and limit the applicability of findings from other settings. Therefore, this prospective study was undertaken to provide region-specific evidence on the clinico-epidemiological profile, mortality burden, and factors associated with mortality among paediatric burn patients treated at a tertiary care centre in Western Rajasthan, thereby addressing an important gap in the local literature and informing preventive and clinical management strategies.

## Materials and methods

Study design and setting

This prospective, longitudinal observational study included 100 paediatric burn patients admitted to the burn unit and surgical wards of Mahatma Gandhi Hospital, a tertiary care teaching hospital affiliated with Dr. Sampurnanand Medical College, Jodhpur, Rajasthan. Ethical approval was obtained from the Institutional Ethics Committee of Dr. Sampurnanand Medical College, Jodhpur (approval number: SNMC/IEC/2025/2846-47, approved on 10 March 2025) prior to study commencement. Written informed consent was obtained from parents or legal guardians of all participants prior to enrolment. Assent was obtained from older children whenever appropriate, according to age and understanding. Data were collected over nine months (April-December 2025).

Inclusion criteria

Children aged ≤18 years presenting with acute burn injuries of any extent were eligible for inclusion in the study. Acute burn injuries were defined as burns presenting within seven days of injury.

Exclusion criteria

Patients with healed or chronic burns and those with systemic comorbidities, including diabetes mellitus, obesity, congenital heart disease, hepatic or pulmonary dysfunction, chronic kidney disease, neurological disorders, immunodeficiency, or ongoing immunosuppressive therapy, were excluded from the study.

Sample size and sampling technique

A consecutive sampling method was used. The sample size was calculated for the estimation of a proportion using a 95% confidence level, an expected mortality proportion of 31% based on the findings of Dhopte et al., and an absolute allowable error of 10%. The minimum required sample size was 82 participants [6]. To improve precision and account for potential missing data, 100 eligible paediatric burn patients were ultimately enrolled.

Data collection procedure

Clinical, laboratory, and microbiological data were collected prospectively in real time from admission to discharge or in-hospital death using a structured case report form specifically developed for this study. The form captured demographic details, burn-related characteristics (burn mechanism, anatomical site involved, and TBSA), clinical parameters, urine output trends, treatment details, laboratory values (haemoglobin, serum potassium, and serum sodium), and microbiological findings. The case report form was reviewed by senior faculty members within the department before implementation to ensure the clarity, adequacy, and relevance of the variables collected.

Clinical and microbiological assessment

TBSA was assessed using the Lund and Browder chart, which is considered more accurate for paediatric patients because it accounts for age-related differences in body surface area distribution [17]. Microbiological assessment included culture and sensitivity testing of burn wound specimens collected on day 1 and day 6 of hospitalisation. Wound specimens were collected under aseptic precautions using a sterile swab technique and processed in the institutional microbiology laboratory according to standard microbiological procedures. Oliguria was defined as urine output of <1 mL/kg/hour, while anuria was defined as negligible or absent urine output during the monitoring period as per institutional protocol.

Primary outcome

The primary outcome was in-hospital mortality, defined as death occurring during the index hospital admission for burn injury.

Statistical analysis

Statistical analysis was conducted using MedCalc® Statistical Software version 22.0 (MedCalc Software Ltd., Ostend, Belgium). Continuous variables were expressed as mean±standard deviation (SD) or median with interquartile range (IQR) and categorical variables as frequencies and percentages. Survivor and non-survivor groups were compared using the unpaired t-test for continuous parameters and the chi-square test or Fisher's exact test for categorical variables. Clinically relevant variables and variables identified on univariate analysis were entered into the multivariable logistic regression model to identify independent factors associated with mortality, reported as adjusted odds ratios (ORs) with 95% confidence intervals. The prognostic discrimination of key laboratory and clinical variables was evaluated using receiver operating characteristic (ROC) curve analysis, with optimal cutoff points derived using Youden's index. A p-value of <0.05 was considered statistically significant.

## Results

Table 1 shows that the majority of patients (64/100, 64%) in the paediatric burn group were men, and 70/100 (70%) were between the ages of one and five years. The most prevalent burn mechanism was found to be scald burns (64/100, 64%), and the most often impacted anatomical regions were the head and neck (63/100, 63%). Notably, 35% of the patients developed oliguria within the first 24 hours after arrival, and 50% (50/100) of the children had not received any kind of primary first aid. Total body surface area (TBSA), an indicator of burn severity, predominantly ranged from 10% to 20% (49/100, 49%). More than two-thirds of the patients required hospitalisation for longer than 10 days.

**Table 1:** Demographic, Injury-Related, and Clinical Parameters in Paediatric Burn Patients

Variable	Category	n (%)
Age (years)	<1	4 (4.0%)
	1-5	70 (70.0%)
	6-10	16 (16.0%)
	11-15	7 (7.0%)
	16-18	3 (3.0%)
Gender	Male	64 (64.0%)
	Female	36 (36.0%)
Type of burn agent	Scald	64 (64.0%)
	Flame	28 (28.0%)
	Electric	8 (8.0%)
Burn site involved	Head and neck	63 (63.0%)
	Anterior trunk and limbs	37 (37.0%)
First-aid status	Adequate	13 (13.0%)
	Inadequate	37 (37.0%)
	Not taken	50 (50.0%)
Urine output in the first 24 hours	Oliguria	35 (35.0%)
	Anuria	10 (10.0%)
	Normal	55 (55.0%)
Total body surface area (TBSA)	10%-20%	49 (49.0%)
	21%-30%	25 (25.0%)
	31%-40%	15 (15.0%)
	>40%	11 (11.0%)
Hospital stay duration (days)	<1	1 (1.0%)
	1-5	6 (6.0%)
	6-10	22 (22.0%)
	11-15	39 (39.0%)
	>15	32 (32.0%)

Table 2 shows that haemoglobin levels at admission and 48 hours exhibited significant intergroup variation, with non-survivors presenting lower values (p=0.01 and p=0.009, respectively). The deceased group exhibited a greater frequency of hypokalaemia (p=0.045). Prolonged hospitalisation (>15 days) was exclusively observed in survivors (p=0.011), and a total body surface area above 40% was significantly associated with mortality (p<0.0001). Moreover, non-survivors exhibited a greater isolation of *Pseudomonas aeruginosa *on day 6 (p=0.012).

**Table 2:** Factors Significantly Associated with Mortality SD, standard deviation; TBSA, total body surface area; t, independent samples t-test statistic; χ², chi-square test statistic

Variable	Group	Survived (n=88, 88%)	Died (n=12, 12%)	Test Statistic	P-value
Haemoglobin (admission)	Mean±SD	11.25±2.19	9.24±2.19	t=2.98	0.010
Haemoglobin (48 hours)	Mean±SD	10.01±2.00	7.63±2.58	t=3.07	0.009
Serum potassium	Mean±SD	3.73±0.78	3.26±0.47	t=2.95	0.045
TBSA of >40%	n (%)	3 (3.4%)	8 (66.7%)	χ²=43.16	<0.0001
Hospital stay15 days	n (%)	32 (36.4%)	0 (0.0%)	χ²=6.42	0.011
Bacteriology: *Pseudomonas*	n (%) (day 6)	4 (4.5%)	4 (33.3%)	χ²=11.89	0.012

Of the 100 paediatric burn patients included in the study, 12 died during hospitalisation, resulting in an overall mortality rate of 12% (12/100).

Table 3 shows that multivariate analysis revealed that a total body surface area (TBSA) over 40% was associated with higher odds of mortality (OR=9.60; p=0.001). Notable relationships were observed between elevated mortality risk and reductions in serum potassium levels (OR=1.66 per mEq/L; p=0.044) and haemoglobin levels (OR=1.41 per g/dL; p<0.001). The influence of nosocomial pathogens was shown by the elevated mortality risk (OR=5.20; p=0.023) associated with *Pseudomonas *infection by day 6. A hospital stay of fewer than five days (OR=4.75; p=0.004) was strongly associated with early mortality outcomes. Oliguria/anuria did not reach statistical significance (p=0.054). However, other factors such as age of <5 years, male sex, and the absence of first help were not statistically significant.

**Table 3:** Multivariate Logistic Regression Analysis of Factors Associated With Mortality in Paediatric Burn Patients (N=100) CI, confidence interval; TBSA, total body surface area; g/dL, grams per decilitre; mEq/L, milliequivalents per litre

Variable	Odds Ratio (95% CI)	P-value
Age of <5 years	1.45 (0.52-4.05)	0.472
Male gender	1.23 (0.45-3.33)	0.681
TBSA of >40%	9.60 (2.31-39.87)	0.001
Haemoglobin (per g/dL decrease)	1.41 (1.08-1.84)	<0.001
Serum potassium (per mEq/L decrease)	1.66 (1.01-2.72)	0.044
*Pseudomonas*-positive (day 6)	5.20 (1.24-21.90)	0.023
First aid not given	1.87 (0.67-5.17)	0.229
Oliguria/anuria	2.95 (0.98-8.85)	0.054
Hospital stay of <5 days	4.75 (1.61-13.98)	0.004
Surgical intervention	0.62 (0.21-1.84)	0.381
Burn agent: flame versus scald	1.32 (0.43-4.06)	0.629

**Figure 1:**
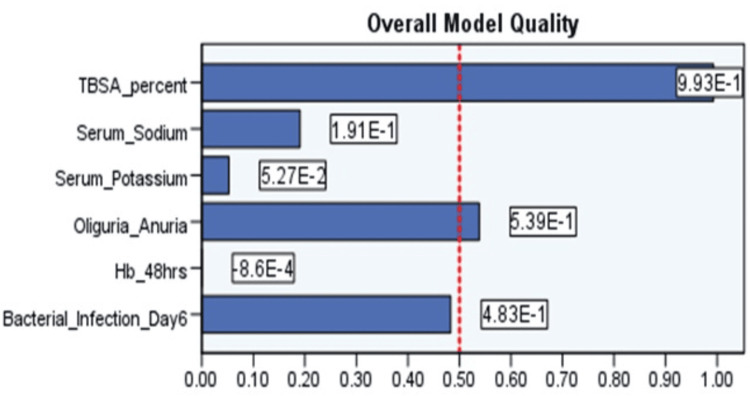
Comparative Contribution of Clinical and Laboratory Parameters to Overall Predictive Model Performance TBSA, total body surface area; Hb, haemoglobin

**Figure 2:**
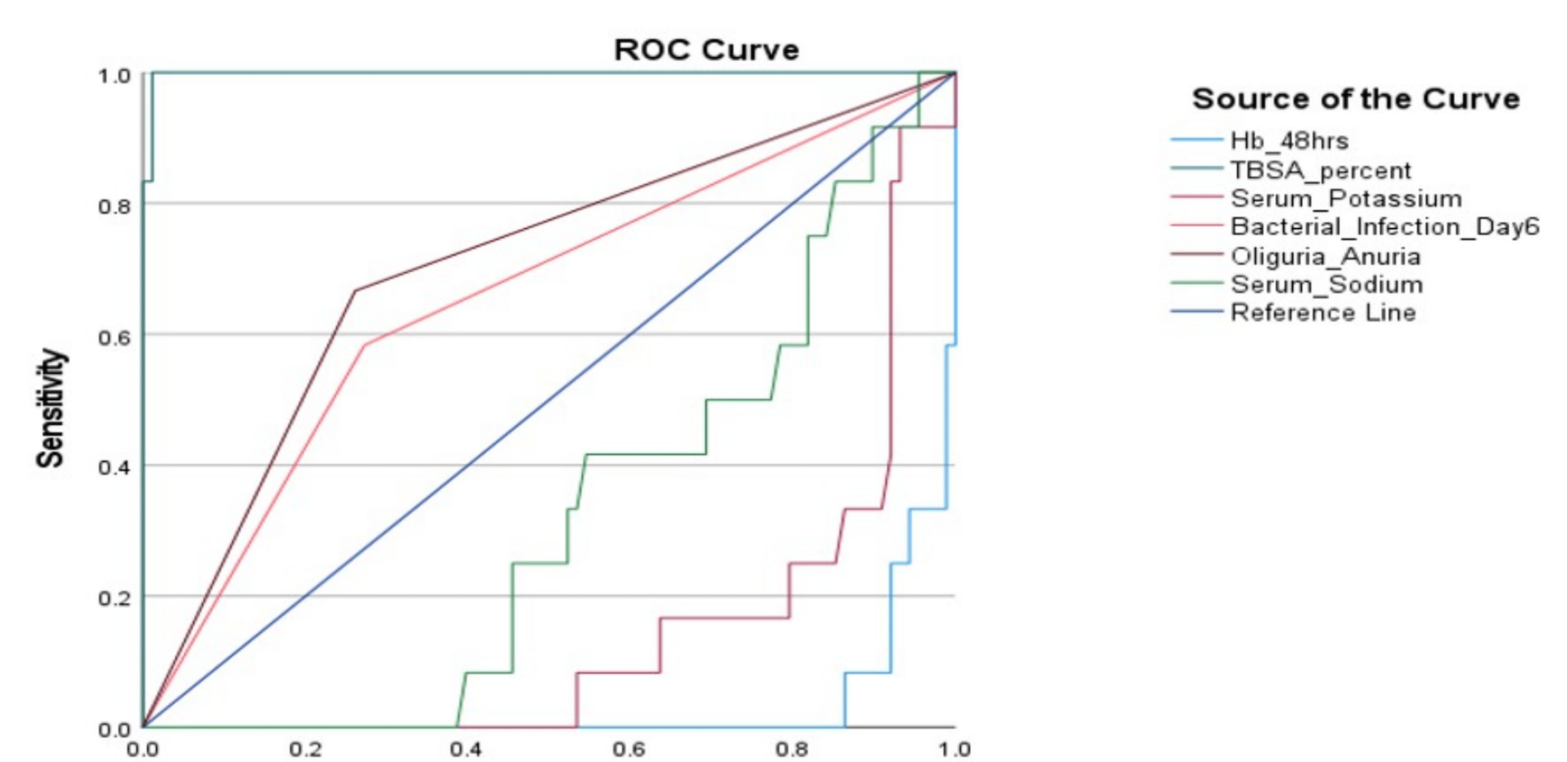
Diagnostic Performance of Clinical and Biochemical Markers for Mortality Prediction in Paediatric Burn Injuries ROC, receiver operating characteristic; Hb, haemoglobin; TBSA, total body surface area

TBSA demonstrated the highest discriminative performance for mortality prediction (area under the curve {AUC}=0.973), with 100% sensitivity and 84.1% specificity at a threshold of 30%. Oliguria/anuria showed moderate discriminatory ability (AUC=0.765). The remaining variables demonstrated lower discriminatory performance when evaluated individually. The discriminative ability of oliguria/anuria was moderate (AUC=0.765; p<0.001), while the bacterial infection on day 6 was not significant. Serum sodium showed poor discriminatory ability for mortality prediction (AUC=0.408) and did not reach statistical significance in ROC analysis (p=0.41). The diagnostic performance of clinical and biochemical factors associated with mortality is summarised in Table 4. The relative contribution of individual clinical and laboratory variables to the predictive model is illustrated in Figure 1. The diagnostic accuracy of major clinical and biochemical markers for mortality prediction in paediatric burn injuries is depicted in Figure 2.

**Table 4:** ROC Curve Analysis for Factors Associated With Mortality in Paediatric Burn Patients AUC, area under the curve; CI, confidence interval; PPV, positive predictive value; NPV, negative predictive value; TBSA, total body surface area; g/dL, grams per decilitre; mEq/L, milliequivalents per litre; mmol/L, millimoles per litre; ROC, receiver operating characteristic

Variable	AUC	95% CI	P-value	Cutoff	Sensitivity (%)	Specificity (%)	PPV (%)	NPV (%)
Haemoglobin (48 hours)	0.256	0.096-0.416	0.003	9.6 g/dL	41.7	39.8	8.6	83.3
Serum potassium	0.321	0.167-0.475	0.023	3.6 mEq/L	33.3	51.1	8.5	84.9
Serum sodium	0.408	0.189-0.627	0.41	132.5 mmol/L	41.7	62.5	13.2	88.8
Bacterial infection (day 6)	0.528	0.353-0.704	0.751	Present	50	55.7	13.3	89.1
Oliguria/anuria	0.765	0.644-0.886	<0.001	Positive	91.7	61.4	24.4	98.2
TBSA (%)	0.973	0.943-1.000	<0.001	30.00%	100	84.1	46.2	100

## Discussion

This prospective, hospital-based study of 100 paediatric burn cases describes the clinical and epidemiological profile of paediatric burn patients in Western Rajasthan. Scald burns were the most common burn mechanism (64/100, 64%), significantly affecting children aged one to five years (70/100, 70%). The overall mortality rate was 12% (n=12). Factors associated with mortality included increased burn severity, reflected by TBSA of >40% in the multivariable analysis; haemoglobin levels below 9.5 g/dL within 48 hours; hypokalaemia (<3.4 mEq/L); and *Pseudomonas aeruginosa* colonisation by the sixth post-burn day. ROC analysis further identified a TBSA threshold of 30% as the optimal cutoff for predicting mortality. Additionally, patients who died had shorter hospital stays, likely reflecting early mortality rather than a prognostic factor. These findings are consistent with multicentre paediatric burn research from both Indian and international populations.

The mortality rate in the present study (12/100, 12%) is comparable to the figure reported by Asefa et al. [11] in Ethiopia and is somewhat lower than that of Purcell et al. in Malawi [13]. Differences in mortality rates across studies may be influenced by variations in sample characteristics, burn severity, TBSA distribution, admission patterns, and healthcare settings. Toma et al. conducted a multi-institutional assessment indicating significant inter-facility variation in paediatric burn mortality within LMIC settings, dependent on the efficiency of pre-hospital care, referring processes, and the availability of specialised burn units [10].

The somewhat decreased mortality in our population may be attributable to the availability of early surgical treatment, compliance with resuscitation using fluid techniques, and regular microbiological monitoring. However, the significant rate of early inpatient fatalities within the initial five days indicates ongoing deficiencies in pre-hospital evaluation and critical care treatment.

By day 6, colonisation by *Pseudomonas aeruginosa *was significantly more prevalent among non-survivors (33.3% {4/12} compared to 4.5% {4/88}), indicating it as a critical modifiable factor influencing outcomes. This corresponds to findings from tertiary Indian centres and African burn units, where *Pseudomonas* is predominant because of its changes to wet wound environments and potential for antibiotic resistance [6,9,12]. The statistics highlight the need for severe infection control, including barrier nursing, early surgical debridement, and pathogen-specific antibiotic management.

Fifty percent of our population exhibited a lack of first aid, a prevalence comparable to the findings of Serrano [8] and Asefa et al. [11] in environments with limited resources. The failure to immediately cool burns with potable running water and provide protective dressings is directly associated with greater burn depth, extended epithelialisation, and more susceptibility to infection [8]. The significant prevalence of head-and-neck involvement (63/100, 63%) in our study is clinically relevant. In addition to the immediate threat of upper airway blockage, these areas are visually and behaviourally delicate. The process of rehabilitation burden is further increased by results presented by Chipp et al., showing a favourable correlation between hypertrophic scar development and delayed epithelial closure in visible anatomical areas [14].

A short hospital stay (less than five days) was greatly associated with mortality (OR: 4.75), according to Asefa et al., who identified short stays as indicators of early in-hospital death rather than faster recovery [11]. In contrast, prolonged inpatient treatment (exceeding 15 days) was uniquely recorded among survivors in our sample, indicating the extensive multidisciplinary care necessary for wound optimisation, nutritional recovery, and psychological support.

Our findings support the association of TBSA, early haematological parameters, electrolyte abnormalities, and infection-related factors with mortality, consistent with paediatric burn literature across multiple settings [5,9-11,13]. The considerable similarity of these mortality-associated factors across LMIC contexts indicates that fundamental management priorities can be adapted; nevertheless, differences in aetiological patterns, microbial ecology, and healthcare infrastructure require region-specific implementation techniques.

This study's findings highlight the need for region-specific treatments targeting both prevention and clinical management from a public health standpoint. Primary prevention must focus on reducing domestic risks, especially those that increase the risk of scald burns in homes with young children. The introduction of complete caregiver education programmes that focus on evidence-based first-aid methods is equally crucial, ensuring prompt and appropriate first-aid measures during the immediate post-burn phase. At the system level, the establishment and implementation of efficient referral pathways may speed the transfer of severe burn cases to specially trained tertiary facilities, thus minimising delays in definitive care. Ultimately, permanent enhancements in outcomes require the implementation of strict infection surveillance systems and compliance with aseptic practices, focused on managing high-mortality organisms such as *Pseudomonas aeruginosa*. When adapted to the cultural and economic contexts of Western Rajasthan and similar low- and middle-income environments, these measures may significantly reduce paediatric burn-related morbidity and mortality.

Clinical recommendations

The findings of the present study have several potential implications for clinical practice in resource-limited settings. Given that scald burns were the predominant mechanism of injury, preventive strategies should focus on reducing household burn hazards and improving caregiver awareness regarding burn prevention. The high proportion of patients who did not receive appropriate first aid highlights an opportunity to strengthen community education on early burn care. In addition, the observed associations of larger total body surface area burns, low haemoglobin levels, hypokalaemia, and *Pseudomonas aeruginosa* colonisation with mortality suggest that the careful early clinical assessment and close monitoring of these parameters may help identify children at an increased risk of adverse outcomes. Strengthening infection prevention practices and ensuring the timely referral of severe burn cases to specialised centres may further contribute to improving clinical outcomes, particularly in regions with limited healthcare resources.

Limitations

This study was conducted at a single tertiary care centre and included a relatively small sample size, which may limit the generalisability of the findings. The observational study design allows the identification of associations but does not establish causal relationships. In addition, certain factors that may influence outcomes, such as burn depth, socioeconomic status, and long-term post-discharge outcomes, were not assessed. Burn depth, an important indicator of burn severity and outcome, was not systematically assessed and therefore could not be included in the analysis. An additional limitation is that the time interval between burn injury and hospital presentation was available for eligibility assessment but was not consistently recorded in sufficient detail for outcome analysis. Despite these limitations, the prospective design and systematic collection of clinical, laboratory, and microbiological data enabled a detailed evaluation of factors associated with mortality in paediatric burn patients. Given the limited number of mortality events, the multivariable findings should be interpreted with caution and require validation in larger cohorts.

## Conclusions

This prospective observational study identified important clinico-epidemiological characteristics and factors associated with mortality among paediatric burn patients at a tertiary care facility in Western Rajasthan. Scald injuries were the predominant burn mechanism and occurred most frequently among children aged one to five years. Greater burn severity, early anaemia, hypokalaemia, and *Pseudomonas aeruginosa *colonisation were associated with mortality. These findings may assist in identifying high-risk patients and support targeted preventive and clinical management strategies to improve outcomes in paediatric burn care. Strengthening caregiver education, early clinical assessment, infection control practices, and timely referral pathways may further contribute to reducing burn-related morbidity and mortality in resource-constrained settings.
